# Definition of a core set of quality indicators for the assessment of HIV/AIDS clinical care: a systematic review

**DOI:** 10.1186/1472-6963-13-236

**Published:** 2013-06-28

**Authors:** Emanuel Catumbela, Victor Certal, Alberto Freitas, Carlos Costa, António Sarmento, Altamiro da Costa Pereira

**Affiliations:** 1 Department of Health Information and Decision Sciences, Faculty of Medicine, University of Porto, Al. Prof. Hernâni Monteiro, Porto, 4200-319, Portugal; 2 Department of Pathology, Faculty of Medicine, Universidade Agostinho Neto, Luanda, Angola; 3 CINTESIS - Center for Research in Health Technologies and Information Systems, University of Porto, Porto, Portugal; 4 National School of Public Health, University Nova de Lisboa, Lisbon, Portugal; 5 Department of Infectious Diseases, Faculty of Medicine, University of Porto, Porto, Portugal

**Keywords:** Performance Measures, Quality Indicator, Infectious Disease

## Abstract

**Background:**

Several organizations and individual authors have been proposing quality indicators for the assessment of clinical care in HIV/AIDS patients. Nevertheless, the definition of a consensual core set of indicators remains controversial and its practical use is largely limited. This study aims not only to identify and characterize these indicators through a systematic literature review but also to propose a parsimonious model based on those most used.

**Methods:**

MEDLINE, SCOPUS, Cochrane databases and ISI Web of Knowledge, as well as official websites of organizations dealing with HIV/AIDS care, were searched for articles and information proposing HIV/AIDS clinical care quality indicators. The ones that are on patient’s perspective and based on services set were excluded. Data extraction, using a predefined data sheet based on Cochrane recommendations, was done by one of the authors while a second author rechecked the extracted data for any inconsistency.

**Results:**

A total of 360 articles were identified in our search query but only 12 of them met the inclusion criteria. We also identified one relevant site. Overall, we identified 65 quality indicators for HIV/AIDS clinical care distributed as following: outcome (n=15) and process-related (n=50) indicators; generic (n=36) and HIV/AIDS disease-specific (n=29) indicators; baseline examinations (n=19), screening (n=9), immunization (n=4), prophylaxis (n=5), HIV monitoring (n=16), and therapy (=12) indicators.

**Conclusions:**

There are several studies that set up HIV clinical care indicators, with only a part of them useful to assess the HIV clinical care. More importantly, HIV/AIDS clinical care indicators need to be valid, reliable and most of all feasible.

## Background

Quality performance or quality of clinical care is the main subject in the health care system and it can be precisely defined. Many studies have been made on this subject [1-3]. The Institute of Medicine defined Quality of care as the degree to which health services for individuals and populations increase the likelihood of desired health outcomes and are consistent with current professional knowledge [3,4]. This assessment can be made by measures that can give us the degree of quality of care. Indicators are defined as a measurement tool that can be used to monitor and evaluate the quality of important governance, management, clinical, and supported functions [5]. They provide a quantitative basis for clinicians, organizations, and planners aiming to achieve improvement in care and in the processes by which patient care is provided [6]. Donabedian and Fleming related indicators to structure, process and outcome [7]. Process indicators assess what the provider did to the patient and if it was well done; it measures the activities and tasks in patient episodes of care. Outcome indicators measure the state of health or events that follow care and that may be affected by health care. In general, either process or outcome may be valid measures of quality. For an outcome to be considered a valid measure of quality, it must be closely related to processes of care that can be manipulated to affect the outcome. Likewise, for a process to be a valid measure of quality, it must be closely related to an outcome that people care about [4]. In many cases, there are multiple factors that contribute to a patient’s survival and health outcome. In those cases, it is useful that outcome measures are adjusted for factors like psychosocial characteristics, lifestyle factors and severity of the illness, if we want to make fair comparisons. Risk adjustment is useful to control confounding factors that might contribute to the outcome indicator [8].

### Quality indicator – development and validation methods

Mainz says that for developing evidence-based clinical indicators, it is necessary to follow certain steps, namely choosing the clinical area to evaluate, organizing the measurement team, providing an overview of existing evidence and practice, selecting clinical indicators and standards, designing measure specification, and performing a pilot test [8].

**Figure 1:**
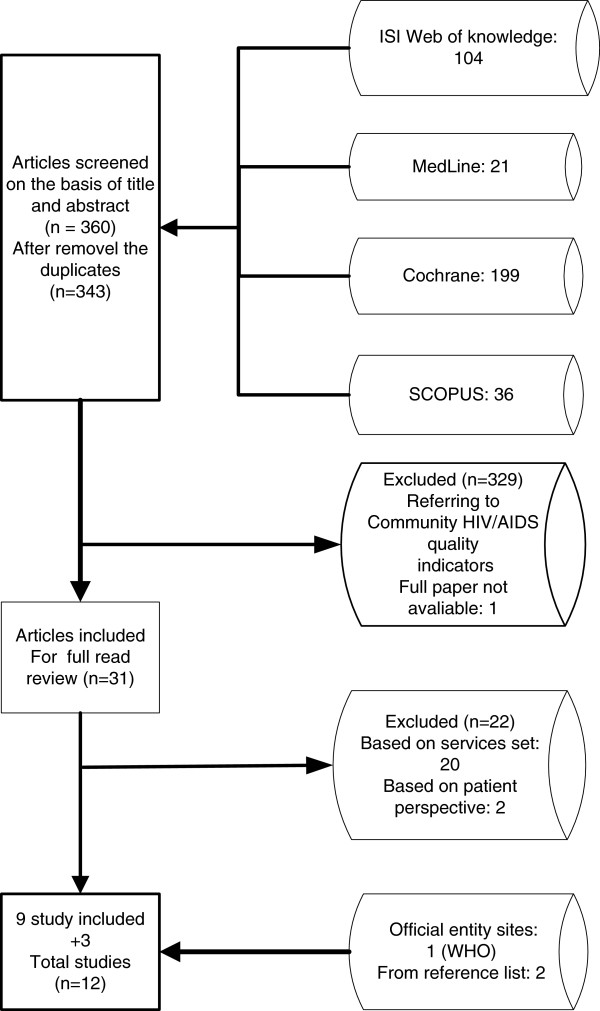
Flow diagram for the search and selection of studies for the systematic review.

The pilot test aims to identify areas that require further specifications of the quality measures. The validation process determines the degree to which an indicator measure, what is intended to measure, that is, whether the results of measurement corresponds to the true state of the phenomenon being measured [8], Figure 1.

### Quality indicators - key characteristics

For each quality indicator it is important to verify if it meets quality requirements such as validity, reliability, relevance and applicability, based evidence [9,10], and the flexibility of obtaining the indicator data [8]. The Agency for Healthcare Research and Quality (AHRQ) uses the following methods for identifying, selecting, and evaluating the quality of the Inpatient Quality Indicators: face validity, precision, minimum bias, construct validity, fosters real quality improvement, and application, mostly for determining the reliability and validity of a quality indicator [11].

The assessment of the quality of care given to HIV/AIDS patients has been a major focus of the HIV/AIDS disease issue since 1990. Agins et al. [12] proposed a model of indicators which assesses clinical care based on tuberculosis (TB) screening, prophylactic therapy and pneumocystis prophylaxis. Mallor et al. [13] proposed a model which assesses the clinical care based on CD4+ count, viral load, as a marker of the evolution of the patient; while Badri and Wood [14] suggested the usefulness of total lymphocyte count in monitoring highly active antiretroviral therapy in resource-limited settings. The New York State Department of Health AIDS Institute [15] uses five indicators to evaluate the quality of clinical care provided to patients with HIV / AIDS, namely: (a) Management of Antiretroviral (ARV) Therapy, (b) Viral Load Monitoring, (c) HIV Intrapartum Prophylaxis, (d) Tuberculosis Screening, and (e) Pelvic Examination. So, we may find different types of indicators used to assess the quality of clinical care given to HIV/AIDS patients.

In this study we performed a systematic review to identify the current existing quality indicators, evidence-based, used for monitoring and evaluating HIV/AIDS impatient and outpatient clinical care, and, based on the clinical relevance and practice utility, we propose a core set of indicators, through observational studies.

## Methods

### Data source

A systematic review (SR) of observational studies on HIV/AIDS quality indicators for clinical care and its development and validation methods, using electronic databases such as MEDLINE (1966–2012 through PubMed), SCOPUS (2004–2012), Cochrane Collaboration database (CENTRAL) (1980–2012) and ISI Web of knowledge ( −2012), without limitations of language. Additional studies were identified by searching reference-lists of articles and on the official institutions sites that work with HIV/AIDS quality indicators.

### Search terms

We used the following search terms and Medical Subject Headings (MeSH) to search observational studies: “observational studies”, “quality indicators”, “validation studies”, “HIV/AIDS”. We developed a search query for each database, and one of the 3 search-queries we set up down can be seen in Table 1. At first, we found all sub heading terms of observational studies and summarized the results. We selected only studies that were carried out on human and excluded those related to animals. After that, we looked for quality indicators and HIV/AIDS studies. This search strategy was based on Cochrane Review recommendations [16].

**Table 1:** MEDLINE search query

**Search**	**Query**	**Items found**
#71	Search #66 AND #70	21
#70	Search #67 OR #68 OR #69	144142
#69	Search HIV/AIDS[Title/Abstract]	17246
#68	Search AIDS[MeSH Terms]	70455
#67	Search HIV [MeSH Terms]	73266
#66	Search #59 AND #62 AND #65	6914
#65	Search #63 OR #64	11513
#64	Search Quality Indicators [Title/Abstract]	2617
#63	Search Quality Indicators, Healthcare [MeSH Terms]	10006
#62	Search #60 NOT # 61	15517434
#61	Search Human [MeSH Terms]	12128326
#60	Search Animal [MeSH Terms]	15794808
#59	Search #31 OR #58	6259659
#58	Search #32 OR #33 OR #34 OR #35 OR #36 OR #37 OR #38 OR #39 OR #40 OR #41 OR #42 OR #43 OR #44 OR #46 OR #47 OR #48 OR #49 OR #50 OR #51 OR #52 OR #53 OR #54 OR #55 OR #56 OR #57	5638100
#57	Search Outcome Assessment (Health Care)[MeSH Terms]	64
#56	Search Outcome Assessment (Health Care)	116277
#55	Search Validation Studies [Publication Type]	54533
#54	Search Evaluation Studies [Publication Type]	162083
#53	Search Case Reports [Publication Type]	1566659
#52	Search Commission on Professional and Hospital Activities[MeSH Terms]	206
#51	Search Commission on Professional and Hospital Activities	282
#50	Search Models, Theoretical[MeSH Terms]	1073230
#49	Search Pilot Projects[Title/Abstract]	453
#48	Search Pilot Projects[MeSH Terms]	69739
#47	Search Research[Title/Abstract]	689721
#46	Search Research[MeSH Terms]	394473
#45	Search Statistics as Topic[MeSH Terms]	1664889
#44	Search Statistics as Topic	1695673
#43	Search Validation Studies as Topic[MeSH Terms]	913
#42	Search Validation Studies as Topic	6755
#41	Search Prospective Studies[Title/Abstract]	17555
#40	Search Prospective Studies[MeSH Terms]	312410
#39	Search Intervention Studies[Title/Abstract]	4683
#38	Search Intervention Studies[MeSH Terms]	5150
#37	Search Twin Studies as Topic[MeSH Terms]	1360
#36	Search Twin Studies as Topic	1784
#35	Search Evaluation Studies as Topic[MeSH Terms]	895895
#34	Search Evaluation Studies as Topic	906192
#33	Search Feasibility Studies[Title/Abstract]	535
#32	Search Feasibility Studies[MeSH Terms]	35041
#31	Search #1 OR #2 OR #3 OR #4 OR #5 OR #6 OR #7 OR #8 OR #9 OR #10 OR #11 OR #12 OR #13 OR #14 OR #15 OR #16 OR #17 OR #18 OR #19 OR #20 OR #21 OR #22 OR #23 OR #24 OR #25 OR #26 OR #27 OR #28 OR #29 OR #30	1509215
#30	Search Longitudinal Studies[Title/Abstract]	9116
#29	Search Longitudinal Studies[MeSH Terms]	757612
#28	Search Retrospective Studies[Title/Abstract]	4389
#27	Search Retrospective Studies[MeSH Terms]	407740
#26	Search Sampling Studies[Title/Abstract]	126
#25	Search Sampling Studies[MeSH Terms]	18424
#24	Search Time and Motion Studies[Title/Abstract]	75
#23	Search Time and Motion Studies[MeSH Terms]	3137
#22	Search Multicenter Studies as Topic[MeSH Terms]	14178
#21	Search Multicenter Studies as Topic	20139
#20	Search Genetic Association Studies[Title/Abstract]	1533
#19	Search Genetic Association Studies[MeSH Terms]	10125
#18	Search Seroepidemiologic Studies[Title/Abstract]	193
#17	Search Seroepidemiologic Studies[MeSH Terms]	15564
#16	Search Follow-Up Studies[Title/Abstract]	8404
#15	Search Follow-Up Studies[MeSH Terms]	438403
#14	Search Cross-Over Studies[Title/Abstract]	284
#13	Search Cross-Over Studies[MeSH Terms]	29156
#12	Search Organizational Case Studies[Title/Abstract]	5
#11	Search Organizational Case Studies[MeSH Terms]	8339
#10	Search Cross-Sectional Studies[Title/Abstract]	3917
#9	Search Cross-Sectional Studies[MeSH Terms]	136893
#8	Search Epidemiologic Studies[Title/Abstract]	13538
#7	Search Epidemiologic Studies[MeSH Terms]	1388630
#6	Search Cohort Studies[Title/Abstract]	8010
#5	Search Cohort Studies[MeSH Terms]	1155017
#4	Search Case–control Studies[Title/Abstract]	8277
#3	Search Case–control Studies [MeSH Terms]	542455
#2	Search observational study[Title/Abstract]	24606
#1	Search observational studies	40232

### Inclusion and exclusion criteria

In this SR we included the primary studies that had met the following criteria, (i) talk about HIV/AIDS clinical care quality indicators, and (ii) set up a conceptual model of quality indicators for HIV/AIDS in clinical care context, or (iii) describe or validate HIV/AIDS clinical care quality indicators.

We excluded studies that (i) are based only on services set, (ii) are based on patient perspective or (iii) did not provide sufficient details in methods and results sections, thus failing to answer research questions.

### Study selection

Eligibility assessment was performed independently by 2 reviewers based on inclusion criteria, and disagreements between reviewers were solved by consensus.

### Data collection processes

We developed a data extraction sheet adapted to observational studies (based on Cochrane Consumers and Communication review Group’s data extraction template), we pilot-tested it on 2 included studies, and refined it accordingly. One reviewer extracted the following data from included studies and the second checked the extracted data: first author, year, publication country and study design, objective, number of clinical indicators and selection criteria. For each indicator: name, type (process or outcome), quality (generic or specific) selection and validation criteria. Disagreements were solved by discussion between the two reviewers. If no agreement could be reached, a third author was planned but it was not necessary. We did not contact any authors for further information.

### Data analysis

For each quality indicator of HIV clinical care some parameters were determined. Similar indicators were grouped together. We classified indicators according to the dimension of quality of care as process (referred to the actions of healthcare providers, such as measuring or screening), or outcome (the results actions of healthcare providers, for instance, non-detectable viral load at 48 treatment week) indicators as described by Donabedian [7]. We also figured out if the American [17] and European [18] guidelines endorsed each indicator. The core set of HIV clinical care indicators was defined by the ones that were simultaneously used in at least two studies and were endorsed by both guidelines.

## Results

### Study selection

The search of MEDLINE, Cochrane, Scopus and ISI web knowledge provided a total of 360 citations. After adjusting for duplicates 343 remained. Of these, 329 studies were discarded after revision as the papers clearly did not met the inclusion criteria. One study, Bennet (1996) [19] was discarded because the full text of the study was not available. The full texts of the remaining 31 citations were examined in more detail. Of those, 22 were excluded for being based on services set (20) and on patient perspective (2). The remaining 9 studies met the inclusion criteria and were included in the systematic review (SR). Two other studies were added to the SR from the reference list. No unpublished relevant studies were obtained. Two papers [20,21] are from the same author and on the same subject and, for that reason, we considered them as one study. As a result, 12 different studies were included for review. The flow diagram for the study selection can be seen in Figure 1.

### Web HIV/AIDS quality indicators search

On the internet, we looked for official sites and only one met the inclusion criteria, namely the Indicator Registry [22] owned by the World Health Organization through United Nations General Assembly Special Session on HIV/AIDS (UNGASS) and other agencies (World Health Organization, UNICEF, the Global Fund to Fight AIDS, Tuberculosis and Malaria, the U.S. President’s Emergency Plan for AIDS Relief, and the UNAIDS Secretariat and guided by the MERG that sets standards for indicators and their use). This registry is a central repository of information on indicators used to track the AIDS epidemic and the national, regional and global response, with 184 indicators.

### Studies characteristics

This systematic review covers a range of 23 years between the first and the last study, published in 2012. Nine studies represent experiences made in the USA [20-28], one in Ethiopia [29], one in Spain [30], one in Thailand [31], and other in Malawi [32]. According to the study design, four are projects that aimed to develop HIV quality indicators [22,23,27,30], three are cross-sectional studies that aimed to assess the clinical care given to HIV/AIDS patients [24,26,29], one is a retrospective cohort [20,21], one is a program evaluation[31], and one is an observational cohort [28]. All studies aimed to assess the quality of a HIV care program, and the last one presents a conceptual model [32] aimed to discuss the validity of indicators within routine programs and their predictive value for ART care. Several indicators were proposed by experts through a panel, and others were taken from guidelines (see Table 2).

**Table 2:** Description of studies by author, year, country, study design, objective, number of clinical care indicators and selection criteria

**Author**	**year**	**Country**	**Study design**	**Objective**	**No. of clinical care indicators**	**Selection criteria**
Mathews et al. [20,21]	1989, 1997	USA	Retrospective cohort	To study patients whose episode of care for pneumocystis pneumonia began on the date of hospital admission, and examine outcomes and processes of care limited to the patient portion of the care episode.	20, 11	Undefined
Wu et al. [23]	2000	USA	Review	To review concepts related to quality of care	17	Expert panel
Asch et al. [24]	2004	USA	Cross-Sectional Study	To evaluate HIV quality of care using a symptom-based, patient-centered framework.	12	Expert panel
Salomon et al. [25]	2005	USA	Cohort study	To assess the impact of patient volume on the quality of care received by AIDS patients within a state’s Medicaid managed system.	15	Guidelines
Wilson at al. [26]	2007	USA	Cross-Sectional Study	To determine whether a selected set of indicators can represent a single overall quality construct.	8	Guidelines
UNGASS Indicator registry [22]	2008	USA	Project	To be a central repository of information on indicators used to track the AIDS epidemic and the national, regional and global response.	9	Guidelines and Expert panel
Alemayehu et al. [29]	2009	Ethiopia	Cross-Sectional Study	To assess the quality of clinical care provided to patients with HIV in Felege Hiwot Referral Hospital.	14	Guidelines and Expert panel
Horberg et al. [27]	2010	USA	Project	To establish a single set of aligned HIV quality measures for care processes and intermediate outcomes for external accountability and individual quality improvement.	17	Guidelines and Expert panel
Hoskins et al. [32]	2010	Malawi	Conceptual modeling	To discuss the challenges of monitoring the progress of the treated population in low-income countries by describing the lack of consensus on indicators, and the burden associated with compiling them.	5	Guidelines and Expert panel
von Wichmann et al. [30]	2010	Spain	Project	To design the present quality of care indicators for persons with HIV/AIDS.	25	Expert panel
Korthuis et al. [28]	2011	USA	Observational cohort	To examine the impact of buprenorphine/naloxone (bup/nx) treatment on quality of HIV care in a multisite cohort of patients with coexisting opioid dependence and HIV infection.	16	Guidelines and Expert panel
Thanprasertsuk et al. [31]	2012	Thailand	Program evaluation	To report experience of HIVQUAL-T implementation in Thailand.	14	Guidelines

### Quality indicators

From the 12 studies, 65 HIV clinical care quality indicators related to process and outcome were retrieved. From these 65 indicators, 29 are HIV/AIDS-specific and 36 are generic indicators; 15 are related to outcome and 50 to process indicators. These indicators cover the following clinical care areas: Baseline evaluation (19), screening for opportunistic diseases and STD (9), immunization (4), prophylaxis (5), HIV monitoring (16), and therapy (12). The boundaries of these clinical areas are not very well delimitated, and therefore some indicators could be easily changed from one to another area (Table 3).

**Table 3:** Quality indicators for the assessment of HIV/AIDS clinical care by name, type, indicators, and selection criteria

**no.**	**Name**	**Type**	**Quality**	**American HIV treatment guidelines**	**European AIDS guidelines**	**Reference**
	A. Baseline evaluation					
1	CD4 cell count measurement	P	S	+	+	[23,25,27,28,30,31]*
2	Appropriate viral load test timing	P	S	+	+	[25,29,31]*
3	Renal basic assessment (Creatinine, Blood urea nitrogen, )	P	G	+	+	[20,31]
4	Complete blood count	P	G	+	+	[20,23,31]*
5	Chemistry analysis (Serum LDH , Sodium, Venous bicarbonate, Hypoxemia ratio, Serum albumin)	P	G	+	-	[24]
6	Proportion of patients with CD4 count greater than 200 cells/ul	O	S	+	+	[29,30]*
7	Bilirubin	O	G	+	+	[24]
8	Admission body mass index	O	G	-	+	[24]
9	Ask about loss of appetite	P	G	-	+	[24]
10	Monitor patient’s weight	P	G	-	+	[22,24]
11	CNS change	O	G	-	+	[21]
12	Complicated cough	O	G	-	-	[24]
13	Diarrhea	O	G	-	-	[24]
14	Heart rate	O	G	-	+	[24]
15	Rales	O	G	-	-	[20]
16	Respiratory rate	O	G	-	-	[21]
17	Lung examination	P	G	+	+	[24]
18	ECG performed in patients on methadone	P	S	-	+	[31]
19	Cardiovascular risk assessment	P	G	-	+	[31]
	B. Screening					
20	Cervical cancer screening (basis and follow up)	P	G	+	+	[26,28,30]*
21	Hepatitis C screening (basis and follow up)	P	G	+	+	[25-27,31]*
22	Hepatitis B testing ever (basis and follow up)	P	G	+	+	[25,27]*
23	Tuberculosis screening	P	G	+	+	[22,23,25-28,30,31]*
24	Gonorrhea/chlamydia screening (at least once)	P	S	-	+	[23,25,27]**
25	Syphilis screening (annually)	P	S	-	+	[25,27,28,30,31]**
26	Injection drug use screening (annually)	P	G	-	+	[23,27,28]**
27	High-risk sexual behavior screening (annually)	P	G	-	+	[23,27,28]**
28	Papanicolau test in last year (for women only)	P	G	-	+	[23,25]
	C. Immunization					
29	Pneumococcal vaccine in previous 6 months if CD4 >200	P	G	-	+	[25,27,28,31]**
30	Influenza vaccination (annually)	P	G	-	+	[26-28]**
31	Hepatitis B vaccination first dose received (if appropriate)	P	G	+	+	[25,27,28,31]*
32	Hepatitis A vaccination	P	G	+	+	[28,31]*
	D. Prophylaxis (patients with <200 lymphocyte CD4)					
33	MAC prophylaxis	P	G	-	-	[23,28]
34	PCP (pneumocystis jiroveci pneumonia) prophylaxis	P	G	-	-	[23,25-28,30,31]
35	Toxoplasmosis prophylaxis	P	G	-	-	[23,31]
36	TB prophylaxis if reactive PPD	P	G	+	-	[25]
37	Cryptococcosis prophylaxis for patients with CD4 <100 cells/ul	P	G	-	-	[30]
	E. HIV Monitoring					
38	Non-detectable HIV viral load at 48 treatment weeks	O	S	+	+	[26,31] *
39	Proportion of patients who received their fist-time CD4 count within 2 weeks after first HIV clinic visit	P	S	-	-	[28,29]
40	Proportion of patients eligible for ART who are currently on ART	P	S	+	+	[29]
41	Proportion of patients on ART for whom adherence is measured on last three visits	P	S	-	-	[29,31]
42	Proportion of patients on NVP who had LFT at least once within 1 month after initiation of NVP-based ART	P	S	-	-	[29]
43	Proportion of patients with previous ARV regimen change for whom reason for change in regime is documented	P	S	+	+	[29,31] *
44	Proportion of patients on ARV with at least 95% (good) reported adherence on last visit	O	S	+	+	[29,32] *
45	Achieving maximal viral control if prescribed ART	O	S	+	+	[27]
46	Achieving maximal viral control if prescribed ART or treatment plan documentation if maximal viral control not achieved.	O	S	+	+	[27]
47	proportion of patients with continued care	P	G	+	+	[29,32] *
48	Visits in three quarters	P	G	+	-	[26,28]
49	HIV counseling and test offered	P	S	+	+	[23,31]*
50	Length of stay in hospital	P	G	-	-	[20]
51	Loss of follow up	P	G	+	-	[31,32]**
52	Percent HIV/AIDS hospital mortality	O	S	-	-	[20]
53	HIV prevalence among pregnant women	P	S	+	+	[22]
	F. Therapy					
54	Appropriately prescribed ART	P	S	+	+	[22,23,25-28,30,31]*
55	Proportion of patients on cotrimoxazole prophylaxis with at least 95% reported adherence on last visit	P	S	-	-	[24,29]
56	Proportion of patients whose CD4 count is <350 cell/ul who are currently on cotrimoxazole prophylactic therapy	P	S	-	-	[29]
57	In patients with at most CD4 count <200 cells/microliter Prescribe an antibiotic or admit the patient to the hospital	P	S	-	-	[24]
58	Proportions of patients on ART who are started on ART within 2 weeks after clinical eligibility is confirmed	P	S	+	+	[29]
59	Proportion of either bedridden or ambulatory patients who have improvement in functional status	O	S	-	+	[29]
60	Proportion of HIV-positive clients given treatment for latent TB infection	P	S	+	+	[22,30,31]*
61	Proportion of HIV-positive registered TB patients given ART during TB treatment	P	S	+	+	[22]
62	Percent of HIV-positive patients in HIV care or treatment (pre-ART or ART) who started TB treatment	P	S	+	+	[22,30]
63	Patient with HIV receiving Hepatitis C treatment	P	G	+	+	[31]
64	Co-management of Tuberculosis and HIV Treatment	P	S	+	+	[22]
65	Number of HIV-positive pregnant women who received antiretroviral to reduce risk of mother-to-child-transmission	P	S	+	+	[22]

Type of indicator: P – Process (50) and O – Outcome (15); Quality indicator: S – specific (29) and G – generic (36). American and European HIV treatment guidelines: (+) present; (−) absent.

Number of indicators by clinical area: A (19); B (9); C (4); D (5); E (16); F (12).

* Indicators that are in both guidelines and are used in more than one study.

** Indicators that are only in one guideline and are used in more than one study.

### HIV/AIDS quality indicators applicability

We analyzed the HIV/AIDS quality indicators studies and found that some of the HIV clinical indicators pertain to assessing the availability of laboratory facilities [20,21,29], HIV clinical care [25,27-29], and quality of care, looking for a symptom-based framework [24] or for determining whether a selected set of indicators can represent a single overall quality construct [26,32] or mortality risk adjustment approach, according to institutional performance [20].

### Selection and validation methods

All studies refer the use of literature review for searching HIV/AIDS quality indicators. Some were based on national guidelines for the treatment of patients with HIV/AIDS [26,29,31], or on expert HIV clinician panels to identify specific processes of care that a clinician would be expected to perform for HIV patients presenting particular symptoms [23,24]. In one study [20,21] there is no clear description of the methods used to select the quality indicators.

In the validation phase of three studies, the proposed indicators were revised after discussion by physicians who were working on an HIV/AIDS control program as well as HIV care-providing facilities, for their local relevance and retained only the indicators where consensus was achieved [22,27-29,31]. In other six studies there was no reference to validation. The main characteristic of all studies is the quality of care assessment and not the approach to the validity of HIV/AIDS quality indicators.

From the internet search, we identified one official site that has a core set of HIV/AIDS indicators, and we retrieved 22 indicators that met our inclusion criteria. For each indicator we read the definition and what it measures, the data type, and the indicator type level to find out the ones we could use to assess hospital care. Nine quality indicators can be used to assess HIV/AIDS clinical care. Five are related to co-management of HIV and TB, two are related to the care of pregnant woman, and two are related to HIV care itself. Six are process related and 3 are outcome indicators.

To answer the question, “What core indicators are useful to assess HIV clinical care?”, we made a selection process through observation of indicators that are used in more than one study, are not the same or do not assess the same situation, and are endorsed by both American and European guidelines; for the treatment of HIV we found 21 HIV/AIDS quality indicators that can be used to assess the clinical care (Table 4).

**Table 4:** Core indicators proposed for the assessment of the quality of HIV/AIDS clinical care

**no.**	**Name**
	A. Baseline evaluation
1	CD4 cell count measurement
2	Appropriate viral load test timing
4	Complete blood count
6	Proportion of patients with CD4 count greater than 200 cells/ul
	B. Screening
20	Cervical cancer screening (basis and follow up)
21	Hepatitis C screening (basis and follow up)
22	Hepatitis B testing ever (basis and follow up)
23	Tuberculosis screening
24	Gonorrhea/chlamydia screening (at least once)
25	Syphilis screening (annually)
27	High-risk sexual behavior screening (annually)
28	Papanicolau test in last year (for women only)
	C. Immunization
31	Hepatitis B vaccination first dose received (if appropriate)
34	PCP (pneumocystis jiroveci pneumonia) prophylaxis
	E. HIV Monitoring
38	Non-detectable HIV viral load at 48 treatment weeks
43	Proportion of patients with previous ARV regimen change for whom reason for change in regime is documented
44	Proportion of patients on ARV with at least 95% (good) reported adherence on last visit
47	proportion of patients with continued care
49	HIV counseling and test offered
	F. Therapy
54	Appropriately prescribed ART
55	Proportion of patients on cotrimoxazole prophylaxis with at least 95% reported adherence on last visit
60	Proportion of HIV-positive clients given treatment for latent TB infection
62	Percent of HIV-positive patients in HIV care or treatment (pre-ART or ART) who started TB treatment

## Discussion

In this review we wanted to find out what HIV/AIDS quality indicators are used to assess clinical care, their development and validation methods. We found few studies. Most of them are process or outcome indicators and, as Mainz defined, “process denotes what is actually done in giving and receiving care and outcomes measures attempt to describe the effects of care on the patients’ health status and populations” [10]. So, through this review, we have an overview of the most relevant aspects that we have to look at when assessing HIV clinical care. The methods used (guidelines and expert panels) to select them are according to what is described in the literature [8,9,33], nevertheless, little information has been given about their validation methods.

We found 50 process indicators that assess different aspects of HIV/AIDS hospital care. Wollersheim et al. [9] say that there is some conflict between the number of indicators selected and the amount of work which must be spent on recording data. They argue that to achieve a good balance, it is recommended to select about 12 clinical indicators for care process.

Attempting to establish the core set of indicators useful to assess HIV/AIDS clinical care, we compared the quality indicators we got with the American and European guidelines. For some indicators, there is no doubt about their relevance, they are in both guidelines and in more than one study, for instance, CD4 count cell, viral load; for others, the difference is that they are only in one guideline, but this doesn’t make them less important. Indicators like syphilis or gonorrhea screening are also very important even though they are not endorsed by the American guideline. The fact of the matter is that there are specific guidelines for each aspect of HIV care. Another important aspect in this comparison between indicators studies and guidelines is that there are some indicators that are in both guidelines, but are only mentioned by one study. For instance, knowing the “Proportion of patients eligible for ART who are currently on ART” is as important as knowing if they have prescribed appropriate ART. Defining a core set of indicators is a hard task.

In Table 4, we have the core set of indicators we think that can be used to approach HIV/AIDS clinical care. Some specific HIV clinical care indicators used are: CD4 cell count, viral load test, non-detectable HIV viral load at 48 treatment weeks, Hepatitis C screening, Hepatitis B testing, Tuberculosis screening, Hepatitis B vaccination, PCP (pneumocystis jiroveci pneumonia) prophylaxis, adherence to ART, appropriately prescribed ART, and HIV-TB co-treatment. All these measures are considered to be critical elements in the clinical care of individuals with HIV/AIDS [25], and they have clinical utility because they are endorsed by guidelines [17,18]. In the core of indicators that we propose, only 3 of the 23 are outcome indicators. Although it is desirable to obtain outcome indicators it is not always possible, due to: firstly, there are many processes that compete for the same outcome [34]; secondly, due to the time needed to obtain the desired result, surrogate measures are used; thirdly, data limits often dictate the extent of the measures that can be established or are not suitable to every HIV-infected patient. Unless a measure addresses potential pitfalls adequately, the results produced from the measure may be misleading rather than helpful [35].

Some indicators are obtained from a set of steps which includes more than one data element, or are influenced by many factors, such as age or gender (adjustment measures). One of the challenges hospitals face nowadays is the quality of the data they collect [36-38]. Some variables can have missing values, can be biased (for instance, because of their main purpose), or may not be possible to collect, and so adjustments can be difficult. Nevertheless the dissemination of indicators throughout regions and hospitals should help them to understand the key elements of HIV/AIDS care that will be under scrutiny, and thereby encourage hospitals to implement data management systems that facilitate measurement of their HIV/AIDS care process and patient outcomes.

When we analyze the validity of the quality indicators, a common characteristic is that most of the studies assess the quality of care, only one is about the validity of the quality indicator itself. So for each indicator, it is necessary to analyze it in order to find out its reliability, relevance and applicability.

Indicators included in the WHO Indicator and Measurement Registry are set up “on a national basis”; some of them can be applied at a local level, but few of them are to assess the quality of HIV clinical care. These indicators are well defined and are ready to be used by everyone as they are valid and reliable.

### Future works

Many institutions have been using HIV/AIDS quality indicators to assess the quality of clinical care and make a benchmarking between caregivers. The results seem to be good, and improvement of care has been achieved [25,26].

Quality care indicators or clinical care indicators are used in many situations, as the ones described next. (1) To improve the quality of care, for instance Asch at al. [24], used a symptom-based framework and their results suggested that symptom-based measures of quality may measure domains that are distinct from those captured by conventional indicators. The matter is that nowadays, when using TARV, many symptoms are more scarce unless people are run out of the healthcare system. While Thanprasertsuk et al. [31] used the quality indicator to improve the quality of care, they compared one setting before and after using quality indicators, and the improvement of quality of clinical care rose from 0 to 95% of accomplishment. (2) To figure out if the system is delivering clinical care, according to the guidelines. Here we noted that Alemayehu et al. [29], focused their work on assessing the accomplishment of the guidelines through process and outcome measures. They pointed out the need for regular monitoring and improvement of processes and outcomes of care in order to achieve good results. (3) To compare institutions or services (benchmarking). Wilson et al. [26], looked through 69 sites from 30 states to determine whether a selected set of indicators can represent a single overall quality construct. The main result of this study was that it is necessary to define a core set of indicators in order to compare sites fairly.

The next step is to test the measures in real data, to find out the reliability of each indicator and to validate a set core of quality indicators that can be used to assess HIV clinical care and may be used to compare services, hospitals or even countries.

This review had several limitations. Although we found few studies, we made a wide search on the web using multiple terms and databases. Search in gray literature was limited. Studies in languages other than English were not included. Anyway, as far as we know, there is no other published systematic review about HIV/AIDS quality indicators for clinical care assessment, and we summarized here a vast and wide set of information. The fact of the matter is that we include experiences from USA, Ethiopia, Malawi, Spain and Thailand, and a core of organizations that work on the HIV/AIDS field under the WHO umbrella.

## Conclusions

We wanted to identify a core set of indicators that represent optimal care and that will facilitate uniform measurement and benchmarking of the quality of HIV/AIDS clinical care at a local level, and can lead to the establishment of comparative reporting between developed and developing countries.

The main findings of this systematic review are that there are efforts from the USA and Spain to establish a national core set of indicators useful to assess the HIV/AIDS clinical care; the assessment of HIV/AIDS clinical care must take into account the main aspects of baseline assessment, screening, immunization, prophylaxis, HIV monitoring, and therapy. Most of the indicators are process-related, are intended to guide (the good practice) and to evaluate the clinical care provided to HIV/AIDS patients.

## Competing interests

The authors declare that no competing interests exist.

## Authors’ contributions

EC carried out the systematic review, conceived the study, and participated in its design and coordination and drafted the manuscript. VC participated in data collection process. AF participated in the analysis of the results and helped to draft the manuscript. CC participated in the design and coordination of the study. AS participated in the design and coordination of the study. ACP conceived the study, and participated in its design and coordination and helped to draft the manuscript. All authors read and approved the final manuscript.

## Pre-publication history

The pre-publication history for this paper can be accessed here:

http://www.biomedcentral.com/1472-6963/13/236/prepub
